# Multitarget Quantitative PCR Improves Detection and Predicts Cultivability of the Pathogen Burkholderia pseudomallei

**DOI:** 10.1128/AEM.03212-16

**Published:** 2017-03-31

**Authors:** Andre Göhler, Trinh Thanh Trung, Verena Hopf, Christian Kohler, Jörg Hartleib, Vanaporn Wuthiekanun, Sharon J. Peacock, Direk Limmathurotsakul, Apichai Tuanyok, Ivo Steinmetz

**Affiliations:** a Friedrich Loeffler Institute of Medical Microbiology, University Medicine Greifswald, Greifswald, Germany; b Institute of Hygiene, Microbiology and Environmental Medicine, Medical University Graz, Graz, Austria; c Institute of Microbiology and Biotechnology, Vietnam National University, Hanoi, Vietnam; d Institute for Geography and Geology, Ernst-Moritz-Arndt-University Greifswald, Greifswald, Germany; e Mahidol-Oxford Tropical Medicine Research Unit, Faculty of Tropical Medicine, Mahidol University, Bangkok, Thailand; f London School of Hygiene and Tropical Medicine, London, United Kingdom; g Department of Tropical Hygiene, Faculty of Tropical Medicine, Mahidol University, Bangkok, Thailand; h Emerging Pathogens Institute, University of Florida, Gainesville, Florida, USA; FDA Center for Food Safety and Applied Nutrition

**Keywords:** Burkholderia pseudomallei, melioidosis, Thailand, qPCR, rice field, soil

## Abstract

Burkholderia pseudomallei is present in the environment in many parts of the world and causes the often-fatal disease melioidosis. The sensitive detection and quantification of B. pseudomallei in the environment are a prerequisite for assessing the risk of infection. We recently reported the direct detection of B. pseudomallei in soil samples using a quantitative PCR (qPCR) targeting a single type three secretion system 1 (TTSS1) gene. Here, we extend the qPCR-based analysis of B. pseudomallei in soil by validating novel qPCR gene targets selected from a comparative genomic analysis. Two hundred soil samples from two rice paddies in northeast Thailand were evaluated, of which 47% (94/200) were B. pseudomallei culture positive. The TTSS1 qPCR and two novel qPCR assays that targeted open reading frames (ORFs) BPSS0087 and BPSS0745 exhibited detection rates of 76.5% (153/200), 34.5% (69/200), and 74.5% (150/200), respectively. The combination of TTSS1 and BPSS0745 qPCR increased the detection rate to 90% (180/200). Combining the results of the three qPCR assays and the BPSS1187 nested PCR previously published, all 200 samples were positive by at least one PCR assay. Samples positive by either TTSS1 (*n* = 153) or BPSS0745 (*n* = 150) qPCR were more likely to be direct-culture positive, with odds ratios of 4.0 (95% confidence interval [CI], 1.7 to 9.5; *P* < 0.001) and 9.0 (95% CI, 3.1 to 26.4; *P* < 0.001), respectively. High B. pseudomallei genome equivalents correlated with high CFU counts by culture. In conclusion, multitarget qPCR improved the B. pseudomallei detection rate in soil samples and predicted culture positivity. This approach has the potential for use as a sensitive environmental screening method for B. pseudomallei.

**IMPORTANCE** The worldwide environmental distribution of the soil bacterium Burkholderia pseudomallei remains to be determined. So far, most environmental studies have relied on culture-based approaches to detect this pathogen. Since current culture methods are laborious, are time consuming, and have limited sensitivity, culture-independent and more sensitive methods are needed. In this study, we show that a B. pseudomallei-specific qPCR approach can detect significantly higher numbers of B. pseudomallei-positive soil samples from areas where it is endemic compared with that from culture. The use of multiple independent B. pseudomallei-specific qPCR targets further increased the detection rate of B. pseudomallei compared with that from single targets. Samples with a high molecular B. pseudomallei load were more likely to be culture positive. We conclude that our quantitative multitarget approach might be useful in defining areas where there is a risk of B. pseudomallei infections in different parts of the world.

## INTRODUCTION

Burkholderia pseudomallei causes melioidosis, an infectious disease with high case fatality rates. This bacterium is a tier 1 select agent and a natural inhabitant of soil and surface waters in tropical and subtropical regions where it is endemic (1–5). Melioidosis is acquired through inoculation, aerosols, or ingestion (6, 7). Most clinical cases of melioidosis are reported from northeast (NE) Thailand and northern Australia (8). In NE Thailand, melioidosis is the third most frequent cause of death due to infectious diseases (8). In northern Australia, it is the most common cause of fatal community-acquired pneumonia (9–11). Melioidosis is not restricted to these regions and is known to occur in other parts of Asia, America, and Africa (3, 4, 12, 13), where the epidemiological situation is less well defined. Moreover, a recently published model predicted an alarming number of 165,000 cases of human melioidosis per year worldwide, from which 89,000 people are predicted to die (14). This study illustrates the fact that major regions of the world have never been examined for the occurrence of B. pseudomallei in the environment. Areas where it is highly endemic and where diagnostic microbiology capabilities are not available may go unrecognized, and environmental surveillance for B. pseudomallei can provide an early warning to clinicians and local authorities. Moreover, precise quantitative environmental detection is also fundamental for shedding light on the ecology of this pathogen.

The majority of previous studies have used culture-based approaches to detect and quantify B. pseudomallei from the environment (4, 5, 12, 13, 15, 16). Culture from soil is influenced by numerous factors, including soil sampling depth (17), detachment of bacteria from the soil matrix (18), soil volume, incubation temperature, and the culture media used (12). Guidelines have been published that standardize environmental sampling of B. pseudomallei for culture techniques (15), although there is still a need to define the optima for a number of culture parameters, such as medium composition and incubation temperature, through relevant studies. The current methodology also lacks selectivity and cannot reliably prevent overgrowth of B. pseudomallei by other bacteria, which can lead to false-negative results. Given that culture methods are labor intensive, are time consuming, and have limited sensitivity, a more sensitive molecular screening of environmental samples is needed to complement culture approaches.

In a previous study, we established a protocol for detecting and quantifying B. pseudomallei DNA directly in soil samples using a single-copy fragment of the type three secretion system 1 (TTSS1) as a quantitative PCR (qPCR) target. Using this, we detected significantly higher numbers of B. pseudomallei genome equivalents (GE) than CFU using culture in a number of samples (19). The question remains as to whether qPCR-based detection of B. pseudomallei has the potential to significantly increase the detection rate in culture-negative samples. Here, we describe the development of a molecular assay using novel B. pseudomallei-specific gene targets, in which we compared single- and multitarget-based qPCR approaches for directly detecting and quantifying B. pseudomallei in 200 soil samples from rice paddies in northeast Thailand with previously published cultural data (12). Furthermore, we applied our molecular approach to soil samples from southern Vietnam where B. pseudomallei was isolated from soil more than 16 years ago (20). The combination of multiple qPCR targets increased the detection rate compared with those from single targets in samples with low B. pseudomallei counts. Our results demonstrate that multitarget qPCR screening can be used to predict growth of B. pseudomallei from soil samples.

## RESULTS

### *In silico* identification of novel B. pseudomallei qPCR target regions.

To date, qPCR-based environmental detection of B. pseudomallei has primarily relied on the TTSS1 target. We investigated the value of additional molecular targets for detecting B. pseudomallei in soil. Using whole-genome sequence data, we identified 11 coding sequences (CDS) that were unique to B. pseudomallei. These CDS had no BLAST hits or hits with an E value less than 1e−10 in other tested Burkholderia genomes (data not shown). Initial SYBR green real-time PCR assays were developed based on these gene sequences, and were then tested with a small panel (Biowatch panel) of DNA samples (*n* = 60) from B. pseudomallei and its near-neighbor species. Four conserved regions, BPSL0092, BPSS0087, BPSS0135, and BPSS0745, were proven to be specific to B. pseudomallei. Two of the four gene targets, namely, BPSS0087 and BPSS0745, were selected for qPCR assay development because initial experiments revealed a lower limit of detection (LOD) than BPSL0092 and BPSS0135 (data not shown). Both open reading frames (ORFs) code for hypothetical proteins.

### Development of highly specific duplex qPCR assay using BPSS0087 and BPSS0745.

We established qPCR probe assays targeting the two single-copy genes BPSS0087 and BPSS0745. Primer and probe sets were created and optimized using genomic DNA of B. pseudomallei K96243 (https://www.ncbi.nlm.nih.gov/genome/?term=K96243) (Table 1). Each single assay and the BPSS0087/BPSS0745 duplex assay showed linearity over several orders of magnitude (see Fig. S1 in the supplemental material). The LODs for BPSS0087 and BPSS0745 targets in the duplex assay were ∼7.0 GE and ∼2.5 GE, respectively, which are in a range similar to the LOD of 3 GE of the previously described TTSS1 qPCR. To verify the specificity of the two gene targets, we tested the genomic DNA of 85 B. pseudomallei and 42 non-B. pseudomallei isolates from different origins (Table 2; see also Table S2) using the BPSS0087/BPSS0745 duplex qPCR and compared the results to those from the TTSS1 assay. All B. pseudomallei were identified and no false-positive amplification products were detected in the non-B. pseudomallei strains using each of the three qPCR assays. Moreover, spiking experiments revealed that neither humic acids nor artificially added soil DNA had an inhibitory effect on assay performance (see Fig. S4 and S5).

**TABLE 1:** Oligonucleotides used in this study

Purpose	Primer or probe	Oligonucleotide sequence^*a*^ (5′ to 3′)	Concn (nM)	Amplified DNA fragment and length	Reference or source
qPCR target Bacteria	16S forward	TGGAGCATGTGGTTTAATTCGA	250	16S rRNA, 159 bp	41
	16S reverse	TGCGGGACTTAACCCAACA	100		
	16S probe	FAM-CACGAGCTGACGACARCCATGCA-BHQ1	100		
Detection of PCR inhibitors	IAC2 forward	GAATTCGCCCTTATTAGCCGAC	400	Insertion sequence of pCR2.1-IAC, 166 bp	19
	IAC2 reverse	GGAATTCGCCCTTTAATGCGC	400		
	CMV3 probe	HEX-TGATCGGCGTTATCGCGTTCTTGATC-BHQ2	260		
qPCR targets for B. pseudomallei	BpTT4176 forward	CGTCTCTATACTGTCGAGCAATCG	400	TTSS 1 gene, 5′ region (−82 to +34) of BPSS1407/*sctD*, 115 bp	42
	BpTT4290 reverse	CGTGCACACCGGTCAGTATC	400		
	BpTT4208 probe	FAM-CCGGAATCTGGATCACCACCACTTTCC-BHQ1	260		
	BPSS0087-Lfw	GAATGCGTGCGCGAGCA	500	BPSS0087 coding region,^*b*^ 88 bp	This study
	BPSS0087-Brev	CTCGGCCGGTCCGGAAT	400		
	BPSS0087-P2	HEX-AGTCGTACGCAGCGCGCG—BHQ2	200		
	BPSS0745-Afw	GCGAGCAAATCCGTCTCTCA	500	BPSS0745 coding region,^*b*^ 132 bp	This study
	BPSS0745-Fsrev	ATGCCAGGGCACATGGCTA	400		
	BPSS0745-P2	FAM-ATCATTCAGGCGGGTGCCGT-BHQ1	200		
BPSS1187 nested PCR for B. pseudomallei	174 outer forward	ACCTTTCGTCGGCATGGTAG	400	BPSS 1187 coding region,^*b*^ 552 bp	19
	725 outer reverse	GCCCGCTTCTGGTCTTTATTC	400		
	8653 inner forward	ATCGAATCAGGGCGTTCAAG	400	BPSS 1187 coding region,^*b*^ 81 bp	43
	8653 inner reverse	CATTCGGTGACGACACGACC	400		
	8653 inner probe	FAM-CGCCGCAAGACGCCATCGTTCAT-BHQ1	260		

a FAM, 6-carboxyfluorescein; HEX, hexachloro-6-carboxyfluorescein; Cy5, indodicarbocyanine; BHQ1, black hole quencher 1; BHQ2, black hole quencher 2.

b Coding sequence denoted for the complete genome sequence of B. pseudomallei K96243 that encodes a hypothetical protein.

**TABLE 2:** Specificities of the qPCR assays^*a*^

Species	No. of samples	No. with positive reaction (%)		
		BPSS0087 qPCR	BPSS0745 qPCR	TTSS1 qPCR
B. pseudomallei	85	85 (100)	85 (100)	85 (100)
B. thailandensis	6	0 (0)	0 (0)	0 (0)
Burkholderia cepacia complex species	30	0 (0)	0 (0)	0 (0)
Non-Burkholderia species^*b*^	6	0 (0)	0 (0)	0 (0)

a 5 ng of each genomic DNA was used.

b Including two Achromobacter species, one Escherichia coli, and three Ralstonia pickettii isolates (see Table S2 in the supplemental material for a complete list).

### Combination of multiple qPCR targets increases the B. pseudomallei detection rates in soil samples from northeast Thailand.

**FIG 1:**
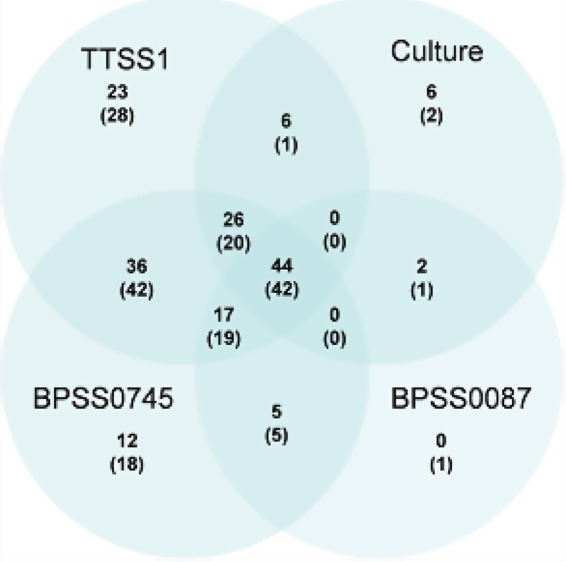
Venn diagram of B. pseudomallei detection in soil samples through a combination of different molecular (qPCR) assays and culture. Ten samples that were only positive by BPSS0745 qPCR and direct culture and one sample that was only positive by BPSS0087 and TTSS1 are not displayed. All six culture-positive and qPCR-negative samples were positive by BPSS1187 nested PCR (19).

We then analyzed 200 soil samples from rice paddies from NE Thailand with the two novel qPCRs and compared the results with those from the established TTSS1 qPCR assay. The same samples were used before to validate different B. pseudomallei cultivation methods as described previously (12). That study by Limmathurotsakul and coworkers used a panel of different culture techniques, including enrichment cultures, and reported 47% (*n* = 94) culture-positive samples, of which 35% (*n* = 70) were positive on direct culture (12). Higher detection rates were observed for the TTSS1 qPCR with 76.5% (*n* = 153) and for the BPSS0745 qPCR with 75% (*n* = 150). Although the analytical sensitivities of the three qPCR assays were comparable (see above and Fig. S1), only 34.5% (*n* = 69) of samples were detected by the BPSS0087 qPCR (Table 3). We found that 90% (*n* = 180) of samples contained BPSS0745 and/or TTSS1 as B. pseudomallei-specific sequences (Fig. 1). When the results from all three qPCR assays were combined, there was only a marginal increase to 91% (*n* = 182) in the overall detection rate, indicating that 97% (67 from 69) of samples that were BPSS0087 positive were also detected by either the BPSS0745 or TTSS1 qPCR (Fig. 1). If the remaining 18 qPCR-negative samples were subjected to the previously described BPSS118*7* nested PCR, the positivity rate reached 100% (Table 1). As pictured in Fig. 1, 61 (30.5%) samples were detected by each of the three qPCR targets, and 130 (65%) samples were detected by at least two qPCR targets.

**TABLE 3:** Detection of B. pseudomallei in 200 soil samples from two Thai rice fields

Result	No. of samples			Sensitivity (% [95% CI])
	Overall	Field 1	Field 2	
Negative	0	0	0	
Positive by culture or at least one PCR assay	200	100	100	100
Culture positive^*a*^	94	47	47	47 (40.2–53.9)
Direct-culture positive^*b*^	70	40	30	35.0 (28.7–41.8)
PCR positive^*c*^	200	100	100	100
qPCR positive^*d*^	182	94	88	91 (86.2–94.3)
TTSS1 positive	153	80	73	76.5 (70.1–81.9)
BPSS0087 positive	69	32	37	34.5 (28.2–41.3)
BPSS0745 positive	150	79	70	75 (68.6–80.5)

a Samples in which B. pseudomallei growth was detected by any culture method used by Limmathurotsakul and colleagues (12).

b Samples in which B. pseudomallei was detected without enrichment according to Limmathurotsakul and colleagues (12).

c Includes all qPCR assays and BPSS1187 nested PCR.

d *B*. pseudomallei-positive samples by any qPCR assay (BPSS0087, BPSS0745, and TTSS1 qPCR).

To verify positive qPCR results from samples with results that were discrepant between different qPCRs (see Fig. S2) and to exclude false-positive detection in single-target positive samples, we cloned and sequenced amplicons from selected samples (see Text S1). We found that sequenced amplicons of either BPSS0087, BPSS0745, or TTSS1 from qPCR-positive samples clearly indicate B. pseudomallei sequences (Text S1). However, we also found evidence that some samples contained yet unknown sequences where primers but not the probes for TTSS1 and BPSS0745 bound (or formed dimers), and thereby either might lower the sensitivity for the respective target or might even lead to false-negative results. These findings are likely to explain the different GE numbers determined by the three qPCR targets in some samples (Fig. S2) and the increased detection rate observed using multiple qPCR targets (Fig. 1).

**FIG 2:**
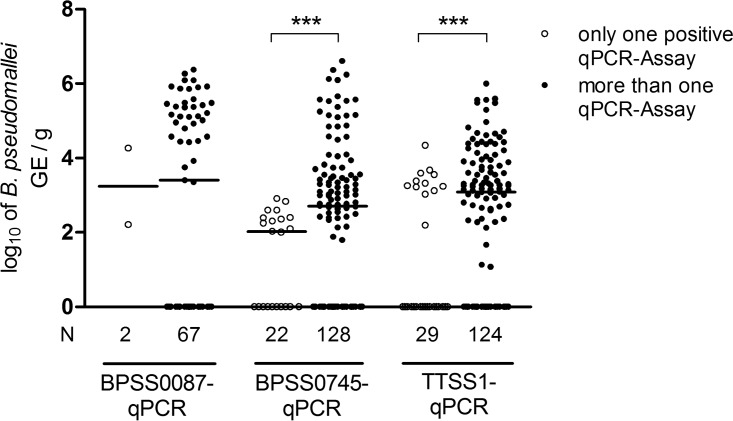
Comparison of B. pseudomallei GE in soil samples with only one and more than one positive qPCR assay(s). Scatter plot (with medians) comparison of log_10_-transformed GE as B. pseudomallei count per gram soil. B. pseudomallei GE of qPCR-positive samples were grouped (for each assay) in samples with one or more positive qPCR assays. Samples with a value of zero are the result of positive and negative subsamples in single qPCRs (see Materials and Methods), leading to a median of zero. N denotes the number of samples per group. ***, *P* < 0.001 as determined by a Mann-Whitney U test.

**FIG 3:**
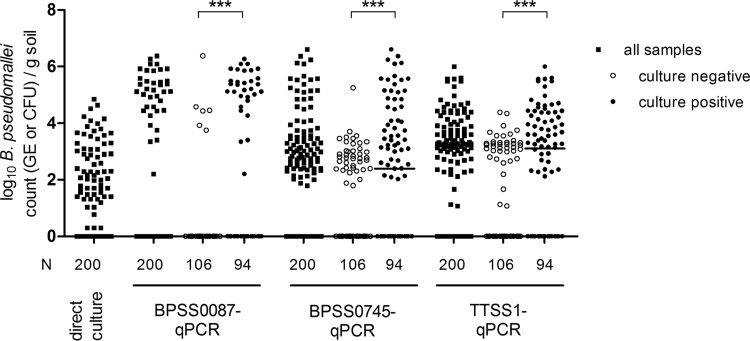
Comparison of B. pseudomallei GE detected by the various assays of culture-positive and -negative samples. Scatter plot (with medians) comparison shows log_10_-transformed B. pseudomallei CFU per gram soil as determined by direct culture and log_10_-transformed B. pseudomallei GE per gram soil as determined by qPCR assays. N denotes number of samples per group. ***, *P* < 0.001 by a Mann-Whitney U test.

The odds of BPSS0087, BPSS0745, and TTSS1 qPCR-positive samples also being direct-culture positive were higher than qPCR-negative samples based on odds ratios (OR) of 6.4 (95% confidence interval [CI], 3.3 to 12.1; *P* < 0.001), 9 (95% CI, 3.1 to 26.4; *P* < 0.001), and 4 (95% CI, 1.7 to 9.5; *P* < 0.001), respectively. In other words, a sample being positive by qPCR was more likely to be direct-culture positive. Samples being positive by at least two qPCR assays (*n* = 130) were 7.1 times more likely to be direct-culture positive (95% CI, 3.1 to 15.9; *P* < 0.001) and 2.2 times more likely to show B. pseudomallei growth, including enrichment (95% CI, 1.2 to 4.1; *P* = 0.009) than samples with one positive or all negative qPCR-assay(s) (see Table S5). This indicates that single-target-positive samples have lower B. pseudomallei GE counts compared with those of multiple-target-positive samples (Fig. 2), which are more likely to be culture positive. Conversely, direct-culture-negative samples exhibited significantly lower B. pseudomallei GE than direct-culture-positive ones (Fig. 3). This effect was significant (Mann-Whitney U test, *P* < 0.001) for each of the qPCR assays. The GE values obtained with the BPSS0087 qPCR exhibited a gap with very few positive samples between 0 and ∼10^4^ GE per g soil (Fig. 3), pointing to a higher LOD in nonartificial DNA mixtures than in pure genomic B. pseudomallei DNA. The B. pseudomallei counts obtained with BPSS0745 or TTSS1 qPCR did not differ significantly (Wilcoxon signed-rank test, *P* = 0.32). These two qPCR assays exhibited significantly higher (Wilcoxon signed-rank test, *P* < 0.001) B. pseudomallei GE counts compared with those from BPSS0087 qPCR and direct culture (B. pseudomallei CFU).

Each of the qPCR assays showed a significant correlation with CFU values as given by Spearman's rho (ρ_sp_) correlation coefficients above 0.46 (see Table S3). Furthermore, the qPCR assays correlated with each other (Fig. S2). As already indicated from Fig. 2 and 3, the BPSS0087 qPCR had decreased sensitivities in samples with a low B. pseudomallei GE compared with those from the other two assays (Fig. S2).

### High molecular B. pseudomallei GE and abundance correlate with cultivability.

**FIG 4:**
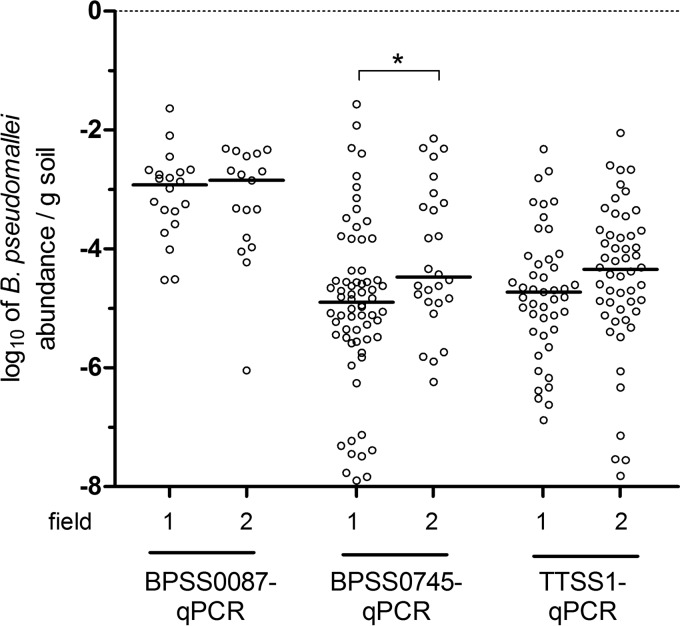
Abundance of B. pseudomallei in single soil samples on fields 1 and 2. The scatter plot (with medians) of B. pseudomallei abundance is given as log_10_ of GE (TTSS1, BPSS0087, and BPSS0745 qPCR) per 16S rRNA gene. *, *P* < 0.05 by a Mann-Whitney U test.

To examine any potential association between the total bacterial load and the presence of B. pseudomallei in the same sample, we determined the B. pseudomallei proportion (abundance). The median of all samples was 9.7 × 10^7^ (range, 1.3 × 10^7^ to 1.9 × 10^10^) bacterial 16S rRNA genes per gram soil. The abundance in one gram of soil, calculated from data of the two sensitive qPCR assays (BPSS00745 and TTSS1), ranged from 0 to 2.7 × 10^−2^ for BPSS0745 qPCR and 0 to 8.86 × 10^−3^ for TTSS1 qPCR B. pseudomallei GE per bacterial 16S rRNA gene (Fig. 4). The median abundance, calculated from these two assays, was 3.15 × 10^−7^ (range, 0 to 7.36 × 10^−3^) B. pseudomallei GE per bacterial 16S rRNA gene.

**FIG 5:**
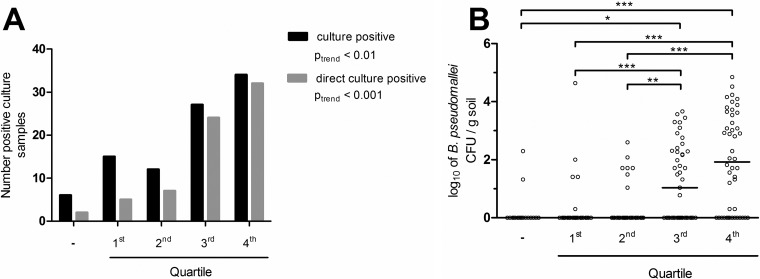
Correlations between (A) culture and direct culture positivity rates and (B) numbers of B. pseudomallei CFU with increasing genome equivalents (GE) of B. pseudomallei. Results were grouped according to calculated means from GE data of BPSS0745 and TTSS1. (B) Data are presented as a scatter plot (with medians); samples were divided between qPCR negative (“-”; *n* = 18) and samples exhibiting positive qPCR assays (*n* = 182), which were grouped in quartiles: 1st, 0 GE/g soil (*n* = 48); 2nd, 0 to ≤4.21 × 10^1^ GE/g soil (*n* = 43); 3rd, 4.21 × 10^1^ to ≤8.37 × 10^2^ GE/g soil (*n* = 45); 4th, >8.37 × 10^2^ GE/g soil (*n* = 46). Trend analysis in panel A was conducted with a chi-square test. *, **, and *** represent *P* values of <0.05, <0.01, and <0.001, respectively, by a Kruskal-Wallis test.

The variations in B. pseudomallei abundance were mainly related to alterations in the B. pseudomallei count rather than changes of total bacterial load. Therefore, we correlated the B. pseudomallei GE to the number of culture-positive samples (Fig. 5A). For instance, only 31% of samples (15 of 48) were culture positive in the first and lowest GE count quartile, whereas 78% of culture-positive samples (36 of 46) were found in samples with the highest GE count (4th quartile). This trend was significant for both culture (*P* for trend < 0.01) and direct culture (*P* for trend < 0.001). Moreover, there was a correlation between the GE, and also for abundance, and the number of B. pseudomallei colonies on agar after direct culture (Fig. 5B; see also Table S4 and Fig. S3). However, a multivariable model is needed to evaluate whether abundance is independently associated with cultivability. In conclusion, the means of the GE calculated from the BPSS0745 and TTSS1 assays serve as predictors for (direct) cultivability in this study.

### B. pseudomallei spatial distribution within rice fields.

**FIG 6:**
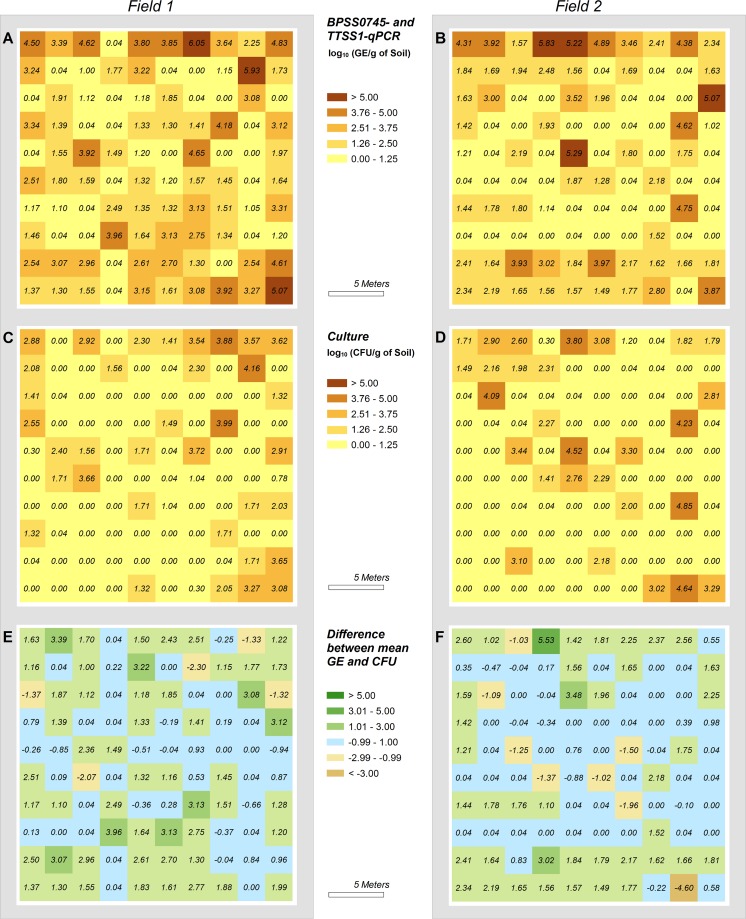
Spatial distributions of B. pseudomallei on both rice fields tested. Field 1 is displayed on panels A, C, and E. Field 2 is displayed on panels B, D, and F. B. pseudomallei counts are given as log_10_ of mean GEs for qPCR (calculated from TTSS1 and BPSS0745) (A, B) or as CFU (for direct-culture method) per gram soil (C, D). Artificial values (0.1 GE or CFU/g soil) were assigned to samples only positive in enrichment culture and to qPCR-positive samples with a calculated median of 0 for differentiation to qPCR- or culture-negative samples. (E, F) Differences between culture and molecular methods are visualized as differences between the log_10_ of the mean GE and CFU values.

The spatial distribution of B. pseudomallei in the two rice fields was visualized for the data obtained from the combination of the two qPCR methods as well as for the culture results (Fig. 6). The molecular and cultural approaches demonstrated similar distribution patterns of B. pseudomallei. As shown in Fig. 3, most samples exhibited significantly higher GE values compared with CFU values. However, 10% of all samples showed CFU values that were >50-fold higher than GE values, as pictured in Fig. 6E and F. It seems likely that a heterogeneity in the microdistribution of B. pseudomallei within single samples (19) is responsible for this phenomenon.

### Application of the multiple qPCR target approach in soil samples from southern Vietnam.

To further test the general applicability of our three-B. pseudomallei-target approach in soil samples from other areas where it is endemic, we analyzed 42 soil samples from 28 sampling sites taken in southern Vietnam. Among those samples, 6 (14.3%) were culture positive and 35 (83.3%) were positive by at least one of the qPCR targets (see Fig. S6), confirming the higher detection rate as seen with the soil samples from Thailand. Five of the six culture-positive samples were positive for all three of the qPCR targets, whereas one sample was negative by TTSS1 but positive by the other two targets (Table S6 and Fig. S6). These results confirm our previous observation that a single-qPCR-target approach might lead to false-negative results. The B. pseudomallei load varied between 0 and 4.71 × 10^4^ GE/g soil (Table S6). Among the qPCR-positive samples, the median load was approximately 10 times higher in culture-positive samples than in culture-negative ones (Mann-Whitney U test, *P* < 0.001). This observation confirms our results obtained with the Thai soil samples (see above), where a higher molecular B. pseudomallei load was also linked to culture positivity.

## DISCUSSION

Most studies on the environmental prevalence of B. pseudomallei in different regions of the world have relied on culture-based methods (4, 12, 13, 15, 16). However, such methods are laborious and time consuming, especially for large-scale screening. This acts as a barrier to mapping the worldwide distribution of B. pseudomallei (3). Even in single rice fields that are highly culture positive across numerous sampling points, significant numbers of adjacent culture-negative samples can be found (21, 22). Although this might reflect spatial heterogeneity, it may also indicate that current protocols do not reliably detect cultivable B. pseudomallei and/or miss viable but noncultivable states of this pathogen. This suggestion is supported by a recent study from Laos, showing that the molecular detection of B. pseudomallei in enrichment cultures of soil samples was higher than the detection of B. pseudomallei growth in the respective subcultures (23). For large-scale environmental screening, a completely culture-independent and sensitive method is desirable. Such a method might be useful for identifying samples for subsequent targeted attempts to culture B. pseudomallei strains.

In a previous study, we showed that direct TTSS1 target-based qPCR from soil samples detected significantly higher numbers of B. pseudomallei genome equivalents than culture-based CFU (19). This study focused on developing direct molecular B. pseudomallei quantification from soil samples by using a limited number of samples, which were mainly highly culture positive. It remains to be shown that direct qPCR-based detection also leads to increased qualitative sensitivity, namely, an increased detection rate of B. pseudomallei in otherwise culture-negative samples.

The results of our present study clearly show that TTSS1-based qPCR detected significantly higher numbers of B. pseudomallei-positive Thai soil samples (76.5%) than did culture (positivity rate, 47%). Interestingly, the detection rate was further increased to 90% by a combination of the TTSS1 qPCR with the BPSS0745-based qPCR. Our data suggest that this increase is due to the additional detection of samples with low GE counts, which are not reliably detected by a single qPCR assay, whereby samples that were false negative by a single qPCR target became positive using another qPCR target. The variable performance of the single qPCRs and the much higher sensitivity of the multitarget approach compared with that of culture were also confirmed in the 42 soil samples tested from southern Vietnam (Fig. S6 in the supplemental material).

As shown by Price et al. with genomic DNA from pure B. pseudomallei cultures, the TTSS1 qPCR can lead to false-negative results (24). The fact that we found culture-positive TTSS1 qPCR-negative samples from Thailand and Vietnam that were positive by BPSS0745 qPCR or BPSS0087 qPCR clearly shows the usefulness of alternative qPCR targets. Our multiple-target approach is further supported by our findings that individual primers bound to yet unknown sequences of non-B. pseudomallei soil microbes, and thereby most likely lowered the sensitivity of single qPCRs (see Text S1), which might even lead to false-negative results. We observed this potential bias with the recently published B. pseudomallei-specific 122018 assay (24). This assay discriminated between B. pseudomallei and B. thailandensis, but exhibited a significant decrease in sensitivity in the presence of large amounts of B. thailandensis DNA (see Fig. S4). Since B. thailandensis is likely to be encountered in environmental samples of B. pseudomallei from regions where it is endemic, this assay would probably lead to false-negative results. A similar effect with B. thailandensis was not found for the BPSS0087 or the BPSS0745 qPCR assay (see Fig. S5). Another recently published B. pseudomallei-specific assay (25) may also not be useful for environmental studies, since the target gene *wcbG* was shown to be present in the genomic DNA of B. thailandensis E555.

Despite these differences, the overall results of the BPSS0087, BPSS0745, and TTSS1 qPCR were correlated. Moreover, results from each of the qPCR assays correlated with the number of B. pseudomallei detected by direct culture in Thai soil samples. BPSS0745 and TTSS1 PCR B. pseudomallei counts were, on average, 5.6 to 7.2 times higher than CFU values. This value was slightly lower than that from our previous study using the TTSS1 qPCR in which we found 10.6-fold higher GE values compared with CFU values in a limited number of mostly highly culture-positive soil samples (19).

In contrast to the relatively constant total bacterial load observed, the B. pseudomallei counts exhibited a strong variation leading to fluctuating abundances ranging from zero to 0.74% of B. pseudomallei in the two tested rice fields. This uneven spread might reflect a microdistribution due to variable biotic and abiotic conditions, which are known to influence the soil microbiome in rice paddies (26–33). By using molecular and cultural methods, we found that in the two rice fields tested, this observed uneven distribution seems to reflect B. pseudomallei presence rather than cultivability.

Another important outcome of our study is that our molecular assays predicted cultivability of B. pseudomallei in Thai and Vietnamese soils. This was shown for qualitative and quantitative results from Thai soil. Samples that are positive for single qPCR or for a combination are more likely to be culture positive. The higher the molecular abundance of B. pseudomallei that is detected, the more likely it is to isolate B. pseudomallei strains. In conclusion, our sensitive multitarget screening and direct quantification might guide future studies on the ecology of B. pseudomallei in different parts of the world.

## MATERIALS AND METHODS

### Bacterial strains and growth conditions.

All bacterial isolates used in this study are listed in Table S2 in the supplemental material. B. pseudomallei K96243 was used as the reference strain. Isolates were stored at −80°C with 20% (vol/vol) glycerol in tryptic soy broth (TSB), were streaked onto Columbia agar plates supplemented with 5% sheep blood (Becton Dickinson, Heidelberg, Germany), and were incubated overnight under aerobic conditions at 37°C. Fresh cultures grown overnight in Lennox broth or tryptic soy broth (TSB) were harvested for bacterial DNA extraction using a QIAamp DNA purification kit (Qiagen, Hildesheim, Germany).

### *In silico* identification of new B. pseudomallei-specific molecular targets.

To identify new specific gene targets of B. pseudomallei, we generated a list of B. pseudomallei conserved genes using comparative genomic analysis. Thirty available B. pseudomallei genomes were used in this study (see Table S1). All genomes were annotated using myRAST (34), and homologs were grouped on the basis of their amino acid sequence similarity using orthoMCL (35, 36). For each gene, the homolog from K96243 was chosen to represent the homology group. These gene sequences were compared against the genome sequences of other Burkholderia species, such as B. thailandensis (*n* = 17), B. mallei (*n* = 17), B. multivorans (*n* = 4), B. ubonensis (*n* = 4), B. oklahomensis (*n* = 2), and B. thailandensis-like sp. (*n* = 3) (37), to identify B. pseudomallei-specific genes using BLAST.

### Soil samples.

Soil samples from two rice fields were taken in Ubon Ratchathani province, NE Thailand, during March (pre-rainy season) 2011 and consisted of sandy loam (12). Each field was divided into a grid system of 10 by 10 (in total 100) sampling points 2.5 m apart, as described before (12). Aliquots of the same samples were used previously to evaluate a simplified culture method for the isolation of B. pseudomallei (12). Here, we refer to CFU data obtained by direct subculture of a soil suspension as a part of the standard method without enrichment (termed “direct culture”) (12). We use the term “culture positive” for any sample that previously showed B. pseudomallei colonies (12) after either direct culture or any enrichment culture in TBSS-C50 (threonine-basal salt solution plus colistin at 50 mg/liter) broth (38), according to published guidelines (12, 39). A 50-g aliquot of each original 200-g sample was shipped at ambient temperature to the laboratory in Germany for molecular analysis. Ten soil samples were taken around Greifswald, Germany, as negative controls from an area where the bacterium is not endemic to confirm the specificity of the PCR methods and to test for cross-contamination. Moreover, 42 soil samples collected at 28 sampling sites in 13 out of 19 provinces in southern Vietnam were tested. The sampling was part of a nationwide B. pseudomallei environmental surveillance carried out in September 2015 (to be published elsewhere). The distance between two consecutive sites ranged from 6 to 153 km (mean ∼44 km). All samples were collected in rice fields at a 30-cm soil depth. At each sampling site, the distance between two consecutive samples ranged from 20 to 30 m. One to three samples from each site were randomly chosen for testing. Cultures were performed using 10 g of soil in 20 ml of Galimand's broth (40). After 2 days of standing incubation at 40°C, 10-fold serial dilutions of the supernatants were spread on Ashdown agar and incubated at 40°C for 5 days. B. pseudomallei was identified on the basis of its characteristic colony morphology and *recA* sequence analysis.

### Extraction of nucleic acids from soil samples and removal of PCR inhibitors.

Upon arrival in Greifswald, Thai soil samples and German negative samples were divided and processed. DNA was extracted at least three independent times per sample using 1-g subsamples. The detailed extraction procedure based on a modified protocol using cetyltrimethylammonium bromide (CTAB) was published before (19). The soil samples from Vietnam (0.5 g) were extracted using the InnuSPEED soil DNA kit (Analytik Jena, Germany). Reextractions of DNA were performed from soil samples that had been maintained at −80°C.

### Standards for qPCR.

As the standard for analysis of the total bacterial load, a 159-bp fragment of the bacterial 16S rRNA gene was cloned into the pSC-B-amp/kan vector (named pSC-16SB), as described elsewhere (41). The inhibition control plasmid pCR2.1-IAC was described before (19) and consisted of an artificial synthesized DNA fragment of 135 bp within a pCR2.1 plasmid (MWG-Biotech, Ebersberg, Germany), exhibiting the same thermal conditions as the TTSS1 target amplification. Each qPCR assay (BPSS0087, BPSS0745, and TTSS1 qPCR) included a standard curve made by serial dilutions of B. pseudomallei K96243 gDNA over five orders of magnitude to ensure and control optimal qPCR efficiencies and to calculate the copy number of the respective target. Since these assays target a single-copy region, the number of amplicon copies can directly be converted into genome equivalents (GE) (19). Bacterial loads of the samples were calculated by using the number of 16S rRNA genes as described previously (41).

### qPCR for determination of inhibition, bacterial load, and B. pseudomallei.

Regions coding for BPSS0087 and BPSS0745 were selected as qPCR targets (see Results). To create a qPCR duplex assay for BPSS0087 and BPSS0745 with optimal conditions, different sets of primers were designed. Final concentrations of the primer/probe combinations are provided in Table 1. Oligonucleotides were purchased from TIB Molbiol (Berlin, Germany), MWG-Eurofins (Ebersberg, Germany), or Microsynth (Balgach, Switzerland). The qPCRs for TTSS1, bacterial load (16S rRNA genes), and inhibition (IAC) were performed as described elsewhere (19, 41). The BPSS0087/BPSS0745 qPCR was performed in parallel as a duplex assay with the following temperature protocol: 50°C for 2 min, 95°C for 10 min, 50 cycles of 95°C for 15 s, 57°C for 30 s, and 72°C for 30 s. The BPSS1187 nested PCR for detecting B. pseudomallei was performed as described for TTSS1-negative samples (19). All qPCRs from duplicates were analyzed using the Light Cycler 480 instrument II (Roche, Mannheim, Germany). For quantifications, the second derivative maximum algorithm was applied (Light Cycler 480 software release 1.5.0). At least six individual standard curves were used per qPCR assay to determine the limit of detection (LOD) according to the work of Price and colleagues (24).

### qPCR quality characteristics and PCR-positive samples.

To ensure sufficient quality of the subsamples, several criteria had to be fulfilled. First, no inhibition of the qPCR was detectable; the difference between the quantification cycle (*C_q_*) value of the sample + 100 copies pCR2.1-IAC and pure pCR2.1-IAC was below a value of 2. Second, more than 10^6^ 16S rRNA genes per PCR were detectable. If one of the three 1-g subsamples per sample did not fulfil these criteria, another 1-g subsample was extracted. A sample was defined as PCR positive if at least one replicate of the three subsamples revealed a *C_q_* value above the limit of detection. Furthermore, all 10 soil sample from a non-endemic region, namely Greifswald, Germany, proved to be negative by B. pseudomallei-specific qPCRs. PCR amplicons from selected samples and qPCR targets were cloned using the Zero blunt cloning kit (Thermo Fisher Scientific, Braunschweig, Germany), were introduced into E. coli DH5a according to the manufacturer's instructions, and were subsequently sequenced.

### Statistical analysis.

The total bacterial loads were used to calculate the percentage of B. pseudomallei within the bacterial community (abundance) after fulfilling the quality criteria (see above). For each single qPCR assay and 1-g subsample, B. pseudomallei GE and the number of rRNA genes per g were calculated as means of the technical duplicates. The medians calculated from the three independent subsamples represented the B. pseudomallei and total bacterial loads per sample and qPCR target, respectively. The abundance of each sample was calculated as the mean GE count of the BPSS0745 and TTSS1 qPCR divided by the bacterial load. GraphPad Prism software version 4.0 for Windows (GraphPad Software, San Diego, CA, USA) was used for calculations and statistical analyses (Wilcoxon signed-rank test, Mann-Whitney U test, chi-square-trend analyses, Kruskal-Wallis test, and correlations by Spearman's rho analyses). For visualizing the distribution of B. pseudomallei, ArcGIS for desktop (version 10.3.1.4959; Environmental Systems Research Institute, Inc.) was used. To differentiate true negative samples from positive samples with a calculated median of 0 GE or CFU in graphical representations, an artificial value of 0.1 CFU or GE per g soil was used.

## Supplementary Material

Supplemental material
