# Supplementary material for: Multitarget Quantitative PCR Improves Detection and Predicts Cultivability of the Pathogen Burkholderia pseudomallei
Source: Appl Environ Microbiol. 2017 Mar 31;83(8):e03212-16. doi: 10.1128/AEM.03212-16 (PMC5377509; doi:10.1128/AEM.03212-16)
Supplement: Supplemental material [file supp_83_8_e03212-16__index.html]

Multitarget Quantitative PCR Improves Detection and Predicts Cultivability of the Pathogen Burkholderia pseudomallei — Supplemental material 

# Multitarget Quantitative PCR Improves Detection and Predicts Cultivability of the Pathogen Burkholderia pseudomallei

## Supplemental material

- Supplemental file 1 -

  Sequencing of PCR amplicons from selected samples (Text S1), sequencing results of cloned amplicons from soil samples using qPCR target primers, *B. pseudomallei* genomes used in this study (Table S1), bacterial strains used in the study (Table S2), Spearman correlation coefficients (Tables S3 and S4), odds ratios (Table S5), overview of the sampling in southern Vietnam (Table S6), detection of *B. pseudomallei* in 42 soil samples from southern Vietnam (Table S7), linearity of *Cq* values (Fig. S1), correlation between qPCR assays (Fig. S2), correlation of culture and direct culture positivity rate and number of *B. pseudomallei* CFU with increasing abundances of *B. pseudomallei* (Fig. S3), influence of genomic DNA of *B. thailandensis* on 122018 qPCR assay (Fig. S4), influence of humic acids, non-*B. pseudomallei* genomic DNA, and soil DNA on the qPCR assays (Fig. S5), and Venn diagram of *B. pseudomallei* detection in Vietnamese soil samples (Fig. S6).

  PDF, 368K
