# Supplementary material for: Multitarget Quantitative PCR Improves Detection and Predicts Cultivability of the Pathogen Burkholderia pseudomallei
Source: Appl Environ Microbiol. 2017 Mar 31;83(8):e03212-16. doi: 10.1128/AEM.03212-16 (PMC5377509; doi:10.1128/AEM.03212-16)
Supplement: Supplemental material [file AEM.03212-16_zam999117781s1.pdf]

Supplementary Text 1: Sequencing of PCR-amplicons from selected samples

We aimed to confirm the presence of *B. pseudomallei*-specific sequences in qPCR-positive soil samples and to investigate if non *B. pseudomallei*-specific amplicons of *BPSS0087-, BPSS0745-* and TTSS1-specific primers exist where the respective probes cannot bind. Touchdown PCRs with the respective primers (see Table 1) were conducted from DNA extracts of selected 1 g subsamples (see table below). Amplicons were cloned into pZero Blunt vector (Thermo Fisher) and transformed into *E. coli* DH5a. Inserts of several selected plasmids per sample and amplicon were sequenced and aligned against sequences of *B. pseudomallei* K96243 using Clustal-Omega (available at www.ebi.ac.uk).

Table Sequencing results of cloned amplicons from soil samples using qPCR target primers

Sample	positive qPCR targtes (GE value per g)*	Amplified and cloned sequences using respective target primers	Alignment	Result[1]
C4.3	*BPSS0087* ($2.24*10^6$)<br>*BPSS0745* ($8.03*10^6$)<br>TTSS1 ($7.89*10^5$)	TTSS1	Amplicon_1 AGGCGTCTCTATACTGTCGAGCAATCGGCGGATATCCGGAATCTGGATCACCACCACTTT<br>Amplicon_2 AGGAGTCTCTATACTGTCGAGCAATCGA--------------------------------<br>TTSS1 ---CGTCTCTATACTGTCGAGCAATCGGCGGATATCCGGAATCTGGATCACCACCACTTT<br>.******************************.<br><br>Amplicon_1 CCGTCCTTGCCCTGGAATGAGACCATGAAGCTGTTACGGATACTGACCGGTGTGCACGCC<br>Amplicon_2 --TACTGACCGGTGTGCACGCC<br>TTSS1 CCGTCCTTGCCCTGGAATGAGACCATGAAGCTGTTACGGATACTGACCGGTGTGCACG--<br>******************	Amplicon_1 is identical to reference; Amplicon_2 shows only primer binding, but no probe binding
C6.2	*BPSS0087* ($9.32*10^4$)<br>*BPSS0745* ($1.7*10^3$)<br>TTSS1 ($1.11*10^4$)	*BPSS0087*	Amplicon CTCGGCCGGTCCGGAATGGAGCGGCTCGCCATCGAGCAGCTTGTGTCGTCGGCGCGC<br>BPSS0087 CTCGGCCGGTCCGGAATGGAGCGGCTCGCCATCGAGCAGTTTGTGTCGTCGGCGCGC<br>*************************************** *****************<br><br>Amplicon GCTGCGTACGACTGTGCTCGCGCACGCATTC<br>BPSS0087 GCTGCGTACGACTGTGCTCGCGCACGCATTC<br>*******************************	99% match, Primer and probes can bind
		BPSS0745	BPSS0745 ---ATGCCAGGGCACATGGCTATCATTCGGCCTTCATTGAAATCGAACGTGCAGCTTGCC<br>Amplicon_3 ---ATGCCAGGGCACATGGCTATCATTCGGCCTTCATTGAAATCGAACGTGCAGCTTGCC<br>Amplicon_1 -AGGTGCCAGGGCACATGGCTAG--------------------------------------<br>Amplicon_2 AGGATGCCAGGGCACATGGCTACTGGTTGT------------------------------<br>.******************<br><br>BPSS0745 GACGAGGGCCCGGAGATCATTCAGGCGGGTGCCGTCAACCGTCTGGACTGGAATGTGAGA<br>Amplicon_3 GACGAGGGCCCGGAGATCATTCAGGCGGGTGCCGTCAACCGTCTGGACTGGAATGTGAGA<br>Amplicon_1 ---GTTTACTTTGTGAGA<br>Amplicon_2 --TGAATGCTCGTTGAGA<br>*: *. : *****<br><br>BPSS0745 GACGGATTTGCTCG<br>Amplicon_3 GACGGATTTGCTCG<br>Amplicon_1 GACGGATTTGCTCG<br>Amplicon_2 GACGGATTTGCTCG<br>**************	Amplicon_1 and Amplicon_2 show similar primer binding, but no probe binding<br>Amplicon_3 is identical to reference, primer and probes can bind
C13.4	*BPSS0745* ($7.6*10^2$)	TTSS1	Amplicon --AGGCTATACTGTCGAGCAATCGGCGGATATCCGGAATCTGGATCACCACCACTTTCCG<br>TTSS1 CGTCTCTATACTGTCGAGCAATCGGCGGATATCCGGAATCTGGATCACCACCACTTTCCG<br>: ***<br><br>Amplicon TCCTTGCCCTGGAATGAGACCATGAAGCTGTTACGGATACTGACCGGTGTGCACGCCT<br>TTSS1 TCCTTGCCCTGGAATGAGACCATGAAGCTGTTACGGATACTGACCGGTGTGCACG---<br>***	99% identical, forward primer with mismatches, probe can bind
C52.3	*BPSS0087* ($1.04*10^5$)<br>*BPSS0745* ($4.21*10^4$)	*BPSS0087*	Amplicon_1 AGGCTCGGCCGGTCCGGAAAGGAGCGGCTCGCCATCGAGCAGTTTGTGTCGTCGGCGCGC<br>BPSS0087 ---CTCGGCCGGTCCGGAATGGAGCGGCTCGCCATCGAGCAGTTTGTGTCGTCGGCGCGC<br>****************:**<br><br>Amplicon_1 GCTGCGTACGACTGTGCTCGCGCACGCATTCCCT<br>BPSS0087 GCTGCGTACGACTGTGCTCGCGCACGCATTC---<br>*******************************	99% identical, primer and probes can bind

<table>
<tr><td>C73.3</td><td>BPSS0087 (5.51*10^{2})</td><td>BPSS0087</td><td><pre>
BPSS0087   AGGCTCGGCCGGTCCGGAATGGAGCGGCTCGCCATCGAGCAGTTTGTGTCGTCGGCGCGC
Amplicon_1   AGGCTCGGCCGGTCCGGAATGGAGCGGCTCGCCATCGAGCAGTTTGTGTCGTCGGCGCGC
Amplicon_2   ---CTCGGCCGGTCCGGAATGGAGCGGCTCGCCATCGAGCAGTTTGTGTCGTCGGCGCGC
                ****

BPSS0087   GCTGCGTACGACTGTGCTCGCGCACGCATTCCCT
Amplicon_1   GCTGCGTACGACTGTGCTCGCGCACGCATTCCCT
Amplicon_2   GCTGCGTACGACTGTGCTCGCGCACGCATTC---
             ****
</pre></td><td>100% identical, primer and probes can bind</td></tr>
<tr><td></td><td></td><td>TTSS1</td><td><pre>
Amplicon_1 AGGCGTCTCTATACTGTCGAGCAATCGCGT------------------------------
TTSS1   ---CGTCTCTATACTGTCGAGCAATCGGCGGATATCCGGAATCTGGATCACCACCACTTT
              ****

Amplicon_1 -----------------------------------CTCGATACTGACCGGTGTGCACGCC
TTSS1   CCGTCCTTGCCCTGGAATGAGACCATGAAGCTGTTACGGATACTGACCGGTGTGCACG--
                                              . ********************
</pre></td><td>only primer bind, but no probe binding</td></tr>
<tr><td>T87.4</td><td>BPSS0745 (1.15*10^{3})</td><td>TTSS1</td><td><pre>
TTSS1   ---CGTCTCTATACTGTCGAGCAATCGGCGGATATCCGGAATCTGGAT-----CACCACC
Amplicon_1 AGGCGTCTCTATACTGTCGAGCAATCG----------CGTCTC-----------------
Amplicon_2 AGGCGTCTCTATACTGTCGAGCAATCGGCGGATATCCCGTCACGCTGTTTGTGATCGGCG
              ************************   *:.:*

TTSS1   ACTTTCCGTCCTTGCCCTGGAATGAGACCATGAAGCTGTTACGGATACTGACCGGTGTGC
Amplicon_1 ---GATACTGACCGGTGTGC
Amplicon_2 TTATCGCGTTCTTGATCG-----CACTTTACGAAGCTGTTACGGATACTGACCGGTGTGC
                                                      ****

TTSS1   ACG---
Amplicon_1 ACG---
Amplicon_2 ACGCCT
           ***
</pre></td><td>only primer bind, but no probe binding</td></tr>
</table>

* GE per g (gram) values calculated from two technical replicates for each subsample; Primer are underlined, Probes are labeled in grey shading; 1) Matches are defined to *B. pseudomallei* K96243 sequence

The comparison of the sequenced fragments after conventional PCR revealed the following:

1. Besides the targeted sequences, the primers of *BPSS0745* and TTSS1 could create non-target PCR-fragments. However, no such clonable amplicons could be found using the *BPSS0087* primers. These *BPSS0745* and TTSS1 amplicons lack sequences that are required for the binding of the *B. pseudomallei*-specific qPCR probe (e.g. C4.3 amplicon 2; C6.2 *BPSS0745*-amplicon 1&2). Consequently, these amplicons cannot be detected in a TaqMan-qPCR assay. This phenomenon was found in samples being either positive or negative for the respective qPCR-target. In positive samples, the binding of the primer to non-target sequences is likely to have a lower sensitivity. In samples being negative for the respective target, this might be responsible for a false-negative result.

2. We could detect *BPSS0087*-, *BPSS0745*- and *TTSS1*-specific sequences in *BPSS0087*-, *BPSS0745*- and *TTSS1*-qPCR-positive samples. Thus, the signals detected by the three qPCRs clearly derived from amplicons generated by the used primers and the respective probes. This was seen in one sequenced *BPSS0745*- (C6.2 amplicon 3) and TTSS1- amplicon (C4.3 amplicon 1) that were identical to several *B. pseudomallei* sequences. All four *BPSS0087*-amplicons were either identical (C73.3 Amplicon 1 & 2) to *B. pseudomallei* sequences in the database or in two cases exhibited one mismatch to *B. pseudomallei* K96243 sequence (sample C6.2 and C52.3 amplicon 1). However, the *BPSS0087*-sequence varies in three nucleotides (~3.4%) within the species of *B. pseudomallei*. Using BLAST, all *BPSS0087*-amplicons (with probe binding site) aligned only to sequences belonging to species *B. pseudomallei*.

3. In one case (sample C13.4), we found a TTSS1-sequence by conventional PCR whereas the TTSS1-qPCR was negative before. This might be due to sequence differences (5 nucleotides) in the 5'-region of the forward-primer and underlines that false negatives can occur with this qPCR assay in environmental samples. Such possible target variations are an additional argument for a multi-target approach for *B. pseudomallei* detection and quantification in environmental samples.

Supplemental Material

Table S1 List of *B. pseudomallei* genomes used in this study

B. pseudomallei	Origin	Source	GenBank#
1106a	Thailand	Clinical	NC_009076.1 & NC_009078.1
1710b	Thailand	Clinical	NC_007434.1 & NC_007435.1
406e	Thailand	Clinical	NZ_CP009298.1 &NZ_CP009297.1
4900CFPatient1	Brazil	Clinical	GCA_000648335.1
Bp22	Singapore	Clinical	NZ_CM001156.1 & NZ_CM001157.1
Gu1909a	Thailand	Clinical	PRJNA179436
K96243	Thailand	Clinical	NC_006350.1 & NC_006351.1
MSHR1655	Australia	Clinical	NZ_CP008780.1 & NZ_CP008779
MSHR1950	PNG	Clinical	PRJNA179436
MSHR305	Australia	Clinical	NC_021877.1 & NC_021884.1
MSHR346	Australia	Clinical	NZ_CP008764.1 & NZ_CP008763.1
MSHR465a	Australia	Clinical	PRJNA179436
MSHR668	Australia	Clinical	NC_007074.1& NC_009075.1
NAU14B6	Australia	Soil	PRJNA179436
NAU20B16	Australia	Soil	NZ_CP004003.1 & NZ_CP004004.1
NAU44A6	Australia	Soil	PRJNA179436
NCTC13178	Australia	Clinical	NZ_CP004001.1 & NZ_CP004002.1
NCTC13179	Australia	Clinical	NC_022659.1 & NC_022658.1
PB08298010	USA	Clinical	NZ_CP009551.1 & NZ_CP009550.1
PHLS9	Pakistan	Clinical	PRJNA179436
Pasteur52237	Vietnam	Clinical	AAHV02000001 – AAHV02000217
RF43BP22	Thailand	Soil	PRJNA179436
RF67BP1	Thailand	Soil	PRJNA179436
RF6BP15	Thailand	Soil	PRJNA179436
NRF80Bp1	Thailand	Soil	PRJNA179436
RF85Bp37	Thailand	Soil	PRJNA179436
RNS3Bp1	Thailand	Soil	PRJNA179436
RNS7Bp6	Thailand	Soil	PRJNA179436
S13	Singapore	Clinical	AAHW02000001 – AAHW02000169
Songkhla34W2	Thailand	Water	PRJNA179436

Table S2 Bacterial strains used in this study

Species	Strain
Achr. xylosoxidans	MEC18
Achr. xylosoxidans	BgE12
B. ambifaria (genomovar VII)	LMG 19182
B. anthina	LMG20980
B. cenocepacia	H111
B. cenocepacia	MAA3
B. cenocepacia	LMG 18830
B. cenocepacia	MBA18
B. cenocepacia	K56-2
B. cepacia	LMG17997
B. contaminans	MAK24
B. diffusa	MBB24
B. dolosa (genomovar VI)	LMG 18941
B. multivorans	LMG 17588
B. multivorans	LMG 16660
B. multivorans	13010
B. pseudomallei	308A
B. pseudomallei	309A
B. pseudomallei	310B
B. pseudomallei	312A
B. pseudomallei	369A
B. pseudomallei	388A
B. pseudomallei	406B
B. pseudomallei	423A
B. pseudomallei	428D
B. pseudomallei	430E
B. pseudomallei	435A
B. pseudomallei	448A
B. pseudomallei	688A
B. pseudomallei	708A
B. pseudomallei	722A
B. pseudomallei	778A
B. pseudomallei	785A
B. pseudomallei	843B
B. pseudomallei	846A
B. pseudomallei	854A
B. pseudomallei	861A
B. pseudomallei	875A
B. pseudomallei	932D
B. pseudomallei	970A
B. pseudomallei	1066A
B. pseudomallei	1069A
B. pseudomallei	1076A
B. pseudomallei	1082A
B. pseudomallei	1099A
B. pseudomallei	1109A
B. pseudomallei	1117A
B. pseudomallei	1119A
B. pseudomallei	1131A
B. pseudomallei	1132A

B. pseudomallei	1142A
B. pseudomallei	1189A
B. pseudomallei	1196A
B. pseudomallei	1210A
B. pseudomallei	1219A
B. pseudomallei	1229A
B. pseudomallei	824aU14
B. pseudomallei	MDS/LC
B. pseudomallei	MK452
B. pseudomallei	MK453
B. pseudomallei	M1900
B. pseudomallei	M1831
B. pseudomallei	K96243
B. pseudomallei	E8
B. pseudomallei	MEA11
B. pseudomallei	MEL21
B. pseudomallei	DM98
B. pseudomallei	WACC56/G1
B. pseudomallei	521
B. pseudomallei	MEF2
B. pseudomallei	770429
B. pseudomallei	BGG22
B. pseudomallei	MEI7
B. pseudomallei	NCTC 8016
B. pseudomallei	56/91
B. pseudomallei	NT08
B. pseudomallei	TAA10
B. pseudomallei	NCTC4846
B. pseudomallei	7665/91
B. pseudomallei	MEH23
B. pseudomallei	MEE3
B. pseudomallei	TAA7
B. pseudomallei	TAA11
B. pseudomallei	BGG16
B. pseudomallei	MEH23
B. pseudomallei	MEE1
B. pseudomallei	MEL16
B. pseudomallei	TAA6
B. pseudomallei	MEC1
B. pseudomallei	BGG19
B. pseudomallei	BGG18
B. pseudomallei	MEF2
B. pseudomallei	MEF1
B. pseudomallei	MEI7
B. pseudomallei	NCTC4845
B. pseudomallei	NCTC10274
B. pseudomallei	NCTC1688
B. pseudomallei	NCTC7383
B. pseudomallei	NCTC10276
B. pseudomallei	NCTC6700
B. pseudomallei	NCTC7431
B. pyrrocinia (genomovar IX)	14191
B. stabilis	LMG7000
B. stabilis	LMG18138
B. stabilis	H118

B. stabilis	LMG14291
B. thailandensis	E201
B. thailandensis	E27
B. thailandensis	E229
B. thailandensis	E236
B. thailandensis	E211
B. thailandensis	E221
B. vietnamensis	LMG10929
B. vietnamensis	MAP6
B. vietnamensis	HGW
B. vietnamensis	LMG10920
B. vietnamensis	MAA5
E. coli	DH5a
Ralstonia picketii	18
Ralstonia picketii	42
Ralstonia picketii	317

Table S3 Spearman correlation (r_{sp}) between CFU from direct culture and genome equivalents GE determined with different assays

Samples	qPCR-assays (or combinations)						
	All qPCR assays (meanGE)	TTSS1/ BPSS0745	TTSS1 /BPSS0087	BPSS0087/ BPSS0745	TTSS1	BPSS0745	BPSS0087
All (N=200)	**0.568**	**0.554**	**0.578**	**0.477**	**0.485**	**0.460**	**0.547**
Only direct culture positive (N=70)	**0.491**	**0.476**	**0.533**	**0.399**	**0.361**	**0.402**	**0.484**

Bold values exhibit p<0.001, the mean GE was calculated for combination of respective assays

Table S4 Spearman correlation (r_{sp}) between CFU and *B. pseudomallei* abundance determined with different assays

Samples	qPCR-assays (or combinations) used for abundance determination						
	All qPCR assays	TTSS1/ BPSS0745	TTSS1 /BPSS0087	BPSS0087/ BPSS0745	TTSS1	BPSS0745	BPSS0087
All (N=200)	**0,576**	**0,560**	**0,571**	**0,465**	**0,475**	**0,447**	**0,540**

Bold values exhibit p<0.001, the mean GE was calculated for combination of respective assays

Table S5 Odds ratio (OR) between qPCR and (direct-) culture

		direct culture				
		positive	negative	total		
TTSS1	positive	63	90	153	OR	4
	negative	7	40	47		
	total	70	130	200		

		direct culture				
		positive	negative	total		
BPSS0087	positive	43	26	69	OR	6,37037
	negative	27	104	131		
	total	70	130	200		

		direct culture				
		positive	negative	total		
BPSS0745	positive	66	84	150	OR	9,035714
	negative	4	46	50		
	total	70	130	200		

		culture				
		positive	negative	total		
TTSS1	positive	76	75	153	OR	1,963333
	negative	16	31	47		
	total	94	106	200		

		culture				
		positive	negative	total		
BPSS0087	positive	46	23	69	OR	3,458333
	negative	48	83	131		
	total	94	106	200		

		culture				
		positive	negative	total		
BPSS0745	positive	80	70	150	OR	2,938776
	negative	14	36	50		
	total	94	106	200		

		culture				
		positive	negative	total		
either 2 assays	positive	70	60	130	OR	2,236111
	negative	24	46	70		
	total	94	106	200		

		Direct culture				
		positive	negative	total		
either 2 assays	positive	62	68	130	OR	7,066176
	negative	8	62	70		
	total	70	130	200		

		culture				
		positive	negative	total		
qPCR*	positive	88	94	182	OR	1,87234
	negative	6	12	18		
	total	94	106	200		

* Any positive qPCR

Table S6 Overview of the sampling in southern Vietnam

Sample Spot			qPCR			
	Sample-Number	Culture positive*	BPSS0087-positive	BPSS0745-positive	TTSS1-positive	*B. pseudomallei* load (GE / g soil)#
1	1.3	No	No	No	Yes	0.1
2	2.4	No	Yes	Yes	Yes	$2.16*10^4$
5	5.2	No	No	Yes	No	0.1
8	8.1	No	Yes	Yes	Yes	$3.89*10^3$
20	20.1	No	No	No	No	0
24	24.1	Yes	Yes	Yes	Yes	$5.06*10^3$
24	24.5	Yes	Yes	Yes	Yes	$1.48*10^4$
25	25.1	No	No	Yes	No	0.1
26	26.1	Yes	Yes	Yes	Yes	$4.12*10^4$
26	26.2	Yes	Yes	Yes	Yes	$4.71*10^4$
31	31.1	No	Yes	Yes	No	0.1
70	70.4	No	No	No	Yes	0.1
71	71.2	No	Yes	Yes	Yes	$9.10*10^2$
72	72.2	No	Yes	No	No	0.1
73	73.1	No	No	Yes	Yes	$1.45*10^3$
73	73.4	No	Yes	No	Yes	0.1
73	73.5	No	No	No	No	0
77	77.1	No	No	Yes	No	$1.71*10^3$
86	86.4	No	Yes	No	No	0.1
86	86.5	No	No	No	Yes	0.1
87	87.5	No	No	No	No	0
94	94.4	No	Yes	No	No	0.1
94	94.2	No	No	No	Yes	0.1
94	94.5	No	Yes	No	No	0.1
95	95.4	No	No	No	No	0
96	96.1	No	No	No	No	0
96	96.5	No	No	No	No	0
97	97.1	No	Yes	No	No	0.1
99	99.2	No	Yes	No	Yes	$1.19*10^3$
99	99.3	No	No	Yes	Yes	$4.32*10^3$
99	99.5	No	No	Yes	No	0.1
102	102.3	No	No	No	Yes	0.1
102	102.4	No	Yes	Yes	Yes	0.1

103	103.2	No	Yes	No	No	0.1
103	103.3	No	Yes	No	Yes	$9.73*10^2$
103	103.5	No	No	No	Yes	0.1
118	118.2	Yes	Yes	Yes	No	0.1
118	118.3	Yes	Yes	Yes	Yes	$1.87*10^4$
126	126.1	No	No	No	No	0
135	135.1	No	No	No	Yes	0.1
138	138.1	No	Yes	No	No	0.1
140	140.1	No	No	Yes	No	0.1
Total (28)	42	6	22	17	21	$0 – 4.71*10^4$

* as determined by 48h cultivation in Galimands broth and Ashdowns agar at 42°C; # Determined as described in material and methods; To differentiate true negative samples from positive samples with a calculated median of '0' GE, an artificial value of 0.1 GE per g soil was used.

Table S7 Detection of *B. pseudomallei* in 42 soil samples from southern Vietnam

	Overall	Sensitivity (%) (95% confidence interval (%))
Negative	7	
Positive by either culture or at least one PCR assay	35	100
Culture-positive[1]	6	17.14 (6.56-33.65)
qPCR-positive[2]	35	100
TTSS1-positive	21	60 (42.11 -76.13)
BPSS0087-positive	22	68.2 (44.92-78.53)
BPSS0745-positive	17	48.57 (31.38-66.01)

[1] the term 'culture-positive' refers to all samples in which *B. pseudomallei* growth could be detected by any culture method; [2] the term 'qPCR-positive' refers to *B. pseudomallei* positive samples by any qPCR reaction (*BPSS0087*, *BPSS0745* and TTSS1-qPCR)

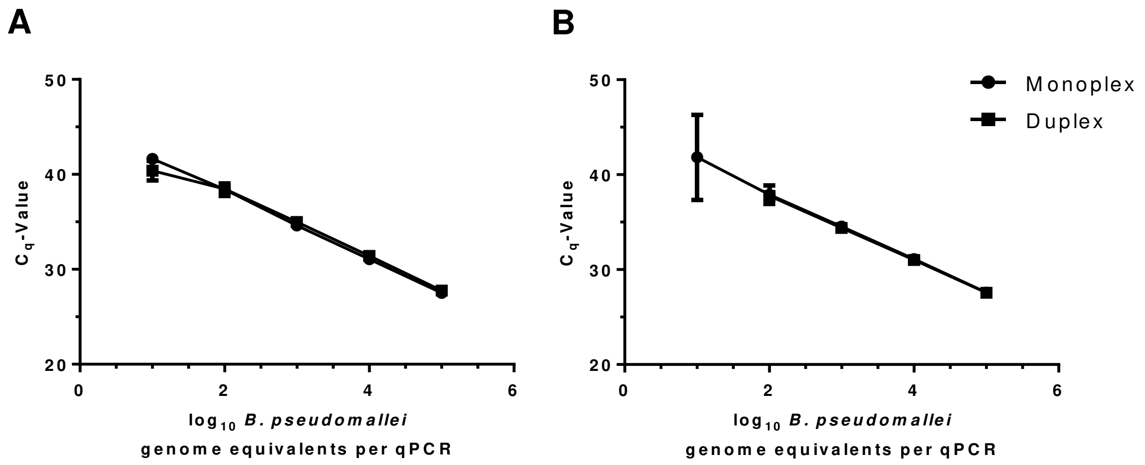

Figure S1 Linearity of C_q-values of the (A) BPSS0087- and (B) BPSS0745- qPCR Monoplex and duplex assay. Data is presented as mean (with SD) of 4 to 6 replicates.

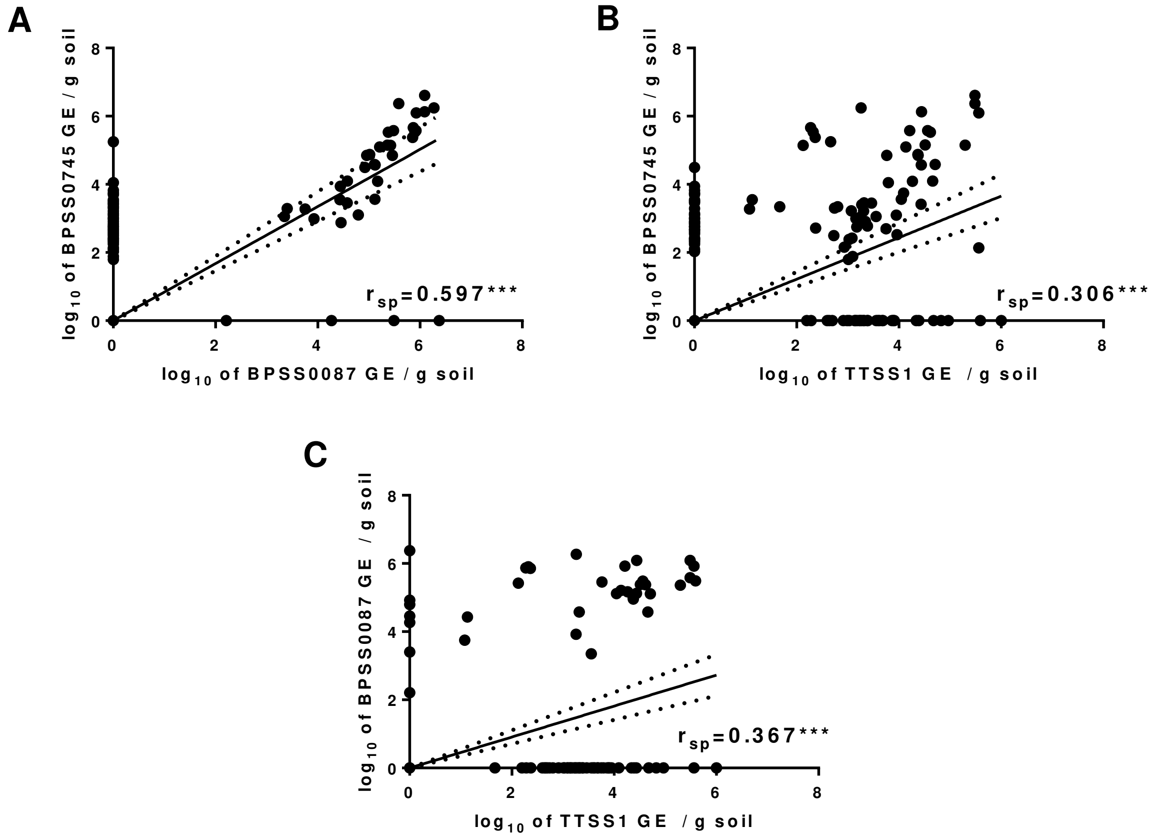

Figure S2 Correlation between qPCR-assays; Correlation of $\log_{10}$ transformed GE values is presented for the qPCR combinations: (A) BPSS0745-/BPSS0087-qPCR; (B) BPSS0745-/TTSS1-qPCR; (C) BPSS0087-/TTSS1-qPCR; *** indicate significance ($p < 0.001$) of spearman correlation (r_{sp})

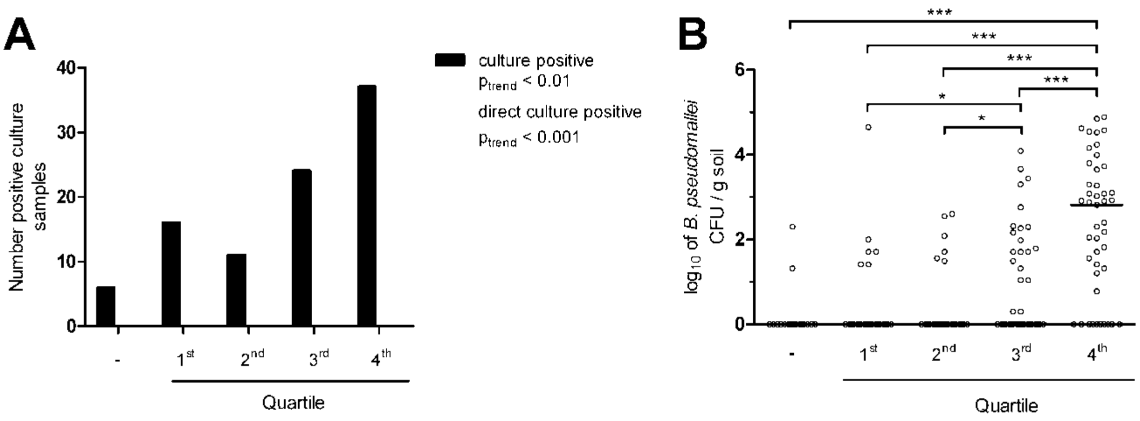

Figure S3 Correlation of (A) culture and direct culture positivity rate and (B) number of *B. pseudomallei* CFU with increasing abundances of *B. pseudomallei*. Results were grouped according to calculated abundance from GE data of BPSS0745 and TTSS1. (B) Data are presented as scatter-plot with median; Samples were divided in qPCR negative ("-"; N=18) and samples exhibiting positive qPCR-assays (N=182), which were grouped in quartiles: 1st 0 to < $9.10101 * 10^{-10}$ GE / 16S rRNA gene (N=49); 2nd 9.10101×10^{-10} to < 4.18726×10^{-7} GE / 16S rRNA gene (N=44); 3rd 4.18726×10^{-7} to < 3.86604×10^{-6} GE / 16S rRNA gene (N=44); 4th 3.86604×10^{-6} to < 7.358818×10^{-3} GE / 16S rRNA gene (N=45). Trend-analysis in panel (A) were conducted with Chi^2-test. */**/*** indicate significance ($p < 0.05/0.01/0.001$) as determined with Kruskal-Wallis-test.

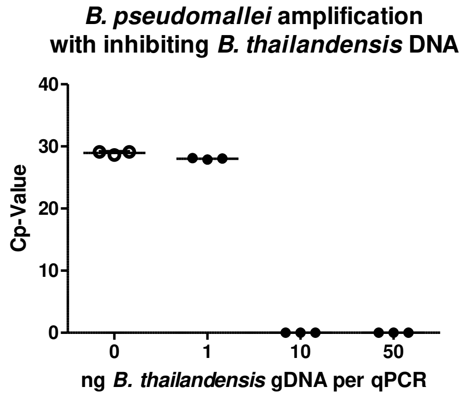

Figure S4 Influence of genomic DNA of *B. thailandensis* on 122018-qPCR-assay. $8*10^4$ Genome copies of *B. pseudomallei* K96243 were amplified with the modified 122018-assay in presence of increasing amounts of *B. thailandensis* E201 gDNA. A clear inhibition was observed when more than 10 ng *B. thailandensis* E201 gDNA per qPCR was used.

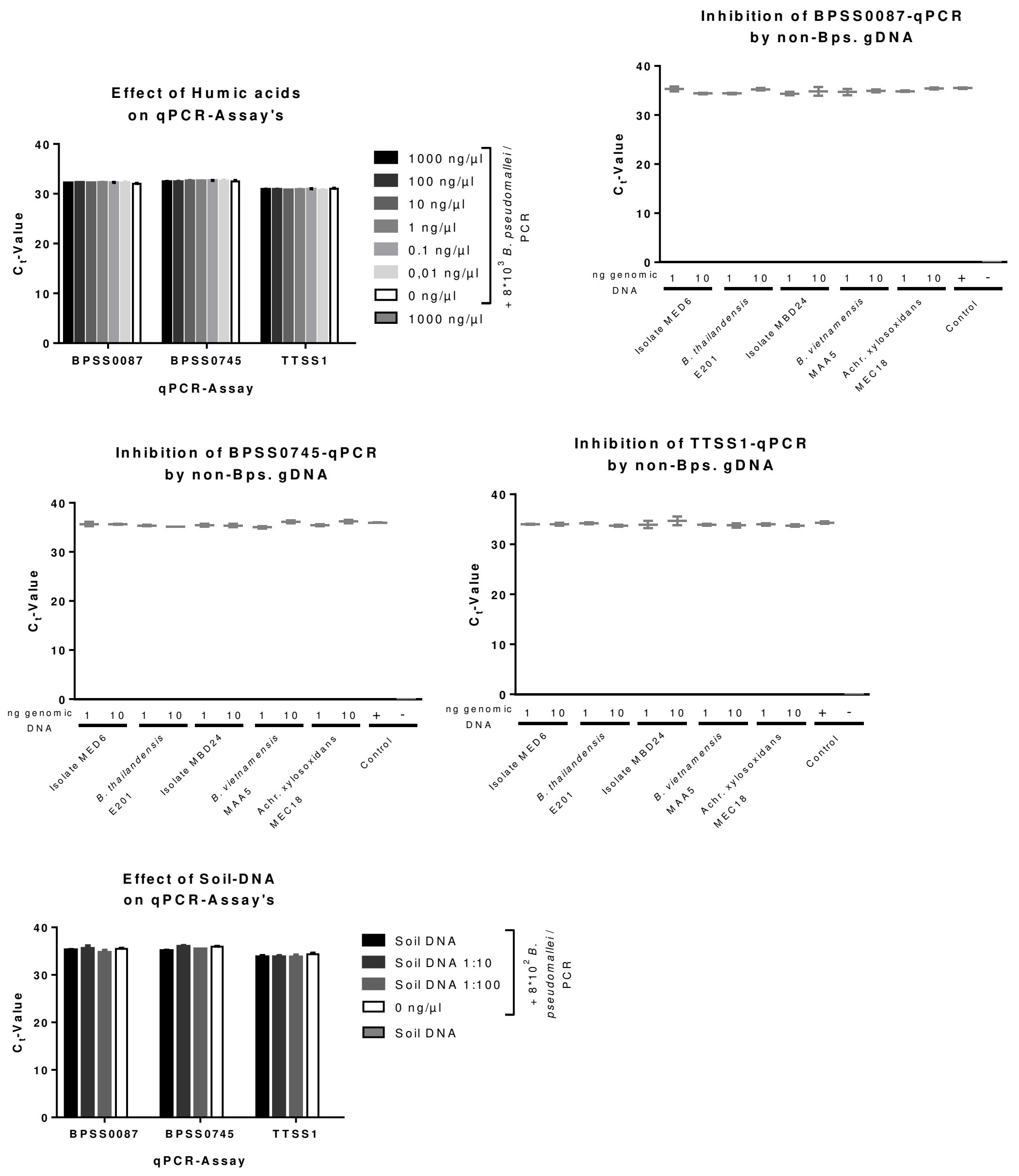

Figure S5 Influence of humic acids, Non-*B. pseudomallei* genomic DNA and soil DNA on the qPCR-assays. 8*10^2 Genome copies of *B. pseudomallei* K96243 were amplified with *BPSS0087*, *BPSS0745* and TTSS1-Assay in presence of increasing amounts of humic acids, genomic DNA from Non-*B. pseudomallei* strains and DNA extracted from Greifswald soil. No inhibition was observed.

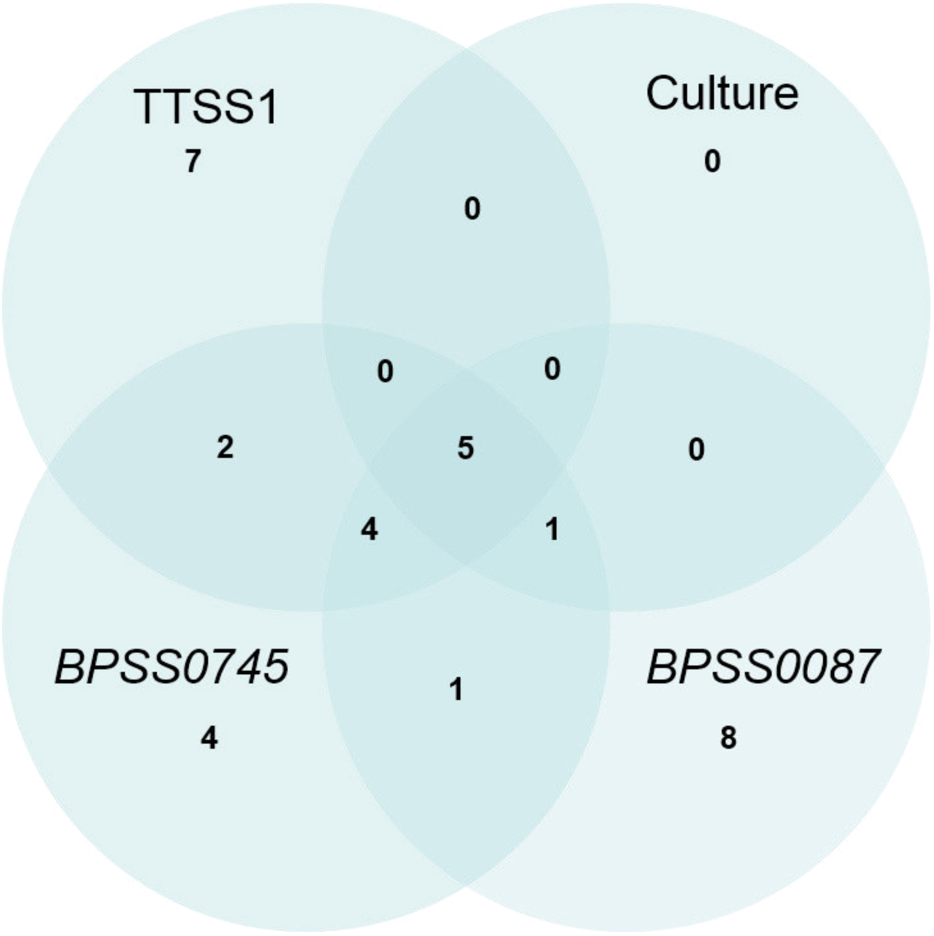

Figure S6 Venn Diagram of *B. pseudomallei* detection in Vietnamese soil samples (N=35) through combination of different molecular (qPCR-) assays and culture. Three samples that were only positive by BPSS0087 and TTSS1 are not displayed.
